# Kinematic stability in cardiac locomotor synchronization during regular walking

**DOI:** 10.3389/fphys.2024.1487465

**Published:** 2024-11-13

**Authors:** Benio Kibushi

**Affiliations:** Graduate School of Human Development and Environment, Kobe University, Kobe, Hyogo, Japan

**Keywords:** maximum lyapunov exponent, acceleration, center of mass, stability, kinematics, electrocardiogram

## Abstract

In locomotion, previous studies have identified a phenomenon known as cardiac locomotor synchronization (CLS), characterized by the phenomenon where heartbeats consistently occur at a specific time within the locomotor cycle. While the physiological significance of CLS is well recognized, its kinematic benefits remain uncertain. Therefore, this study aimed to elucidate the kinematic benefits of CLS under regular walking. Smoothness of walking and local dynamic stability was assessed through the RMS of center of mass acceleration (RMS-CoM_acc_) and maximum Lyapunov exponent (maxL). It was hypothesized that stronger CLS would lead to reduced RMS-CoM_acc_ and maxL. Thirteen participants performed a 10-minute walk at 5.0 km/h. The electrocardiogram and the motion capture data were recorded. To evaluate the CLS, phase coherence (*λ*) between cardiac and walking step rhythm was computed. The high and low-phase coherence was defined as *λ* ≥ 0.1 (*λ*_high_) and *λ* < 0.01 (*λ*_low_); corresponding RMS-CoM_acc_ and maxL values were compared for each state. Although the *λ*_high_ was significantly higher than *λ*_low_, no significant differences in RMS-CoM_acc_ and maxL were observed between the high and low states. The relatively weak CLS observed herein might not have led to a reduction in RMS-CoM_acc_ and maxL. In addition, regular walking speed might be a velocity at which it is challenging to generate intervention effects. Hence, the CLS appears to have negligible impact on the smoothness of walking or local dynamical stability at a 5.0 km/h.

## Introduction

The cardiac locomotor synchronization (CLS) refers to the phenomenon where synchronization occurs between heartbeats and the locomotor cycle. Although the CLS offers physiological advantages (Kirby et al., 1989; Niizeki, 2005; Niizeki and Saitoh, 2014; Takeuchi et al., 2014), the potential contribution of CLS to kinematic stability remains unclear. Further clarifying the kinematic benefits can offer additional insights into the significance of CLS.

Quantitative methods used to assess CLS during walking and running include analyzing the R-wave of heartbeats in conjunction with the timing of heel strikes and muscle activity during gait (Nomura et al., 2006; Takeuchi et al., 2014). CLS refers to the phenomenon where heartbeats consistently occur at a specific time within the locomotor cycle. Both phase synchronization and frequency synchronization have been extensively studied. Unlike frequency synchronization, which aligns only the frequencies of two signals, phase synchronization also aligns their timing, signifying a more robust state of synchronization. This study’s CLS is predicated on phase synchronization. Researches have been shown that CLS is associated with a decrease in oxygen intake during walking (Takeuchi et al., 2014) or CLS occurs by avoiding the peak of intramuscular pressure (Niizeki, 2005). However, synchronization between the systolic part of the cardiac cycle and the locomotor cycle might make it difficult to obtain the physiological benefits of CLS. Synchronizing foot strikes during running with the systolic phase is known to result in higher heart rates than during diastole (Nomura et al., 2006; Constantini et al., 2018). While the physiological aspects of CLS are well recognized, its kinematic benefits remain uncertain. Furthermore, it is worth noting that CLS does not consistently occur but appears to synchronize under specific conditions. Niizeki (2005) found that CLS was only observed when applying 120 mmHg pressure, simulating walking blood flow patterns by using cuff pressure on the thigh (Niizeki, 2005). Nomura et al. (2006) induced CLS by subjecting individuals to an exercise load that elevated heart rate to 160 beats per minute while running (Nomura et al., 2006). Takeuchi et al. (2014) induced CLS with an exercise load that raised the heart rate to 120 beats per minute during walking, ensuring that the heart rate matched step frequency (Takeuchi et al., 2014). However, the walking conditions in this research correspond to fast walking loads. According to Waters et al. (1988), walking speed and heart rate during adult fast walking were 6.62 km/h (male), 5.94 km/h (female), 122.08 beats/min (male), and 127.34 beats/min (female), respectively. Thus, CLS during walking was examined under high exercise loads. Recent reports have described naturally occurring CLS (de Carvalho et al., 2020; Rosato et al., 2024). Specifically, this natural coupling occurs over a wider range of step rates than previously observed in laboratory settings (Rosato et al., 2024), suggesting that CLS could contribute to physiological benefits during regular walking. Although the physiological benefits of CLS are well-documented, it’s uncertain if these benefits improve kinematic aspects like walking stability. Clarifying CLS’s kinematic impact at natural walking speeds could unveil new roles for CLS in regular walking. Understanding the kinematic significance of CLS during regular walking is understudied.

Kinematic stability is one of the kinematic advantages. One proposed method for quantifying the kinematic stability of walking involves measuring the root mean square (RMS) of upper body acceleration (Mizuike et al., 2009; Iosa et al., 2012; Senden et al., 2012). This metric provides insights into the smoothness of walking motions. Previous studies have demonstrated that the RMS of upper body acceleration can efficiently identify instabilities in walking. Research has shown that in healthy young adults, walking under unstable conditions leads to an increase in the RMS of upper body acceleration (Menz et al., 2003). Among elderly individuals, there exists a moderate significant correlation between the RMS of upper body acceleration and a subjective measure of fall risk, such as the Tinetti scale (Senden et al., 2012). Furthermore, there is a notable increase in RMS as stroke severity escalates (Mizuike et al., 2009; Iosa et al., 2012). Thus, upper body acceleration is strongly correlated with unstable walking. Apart from upper body acceleration measurements, the maximum Lyapunov exponent (maxL) was utilized. By quantifying maxL, the local stability of a dynamical system can be evaluated by determining whether the trajectory distances on an attractor diverge or converge. Studies have identified a relationship between maxL and the actual stability of walking (Lockhart and Liu, 2008; Bruijn et al., 2012). Specifically, simulations using walking models and comparisons between elderly individuals with and without fall histories have highlighted a correlation between maxL and fall risk (Lockhart and Liu, 2008; Bruijn et al., 2012). Consequently, maxL is considered a reflection of actual walking instability in research settings. Therefore, the combination of RMS of upper body acceleration and maxL facilitates the examination of the kinematic advantages of walking from the aspects of walking smoothness and local dynamical stability.

The present study investigated the kinematic advantages of CLS during regular walking. Previous studies have shown that preferred walking involves minimizing energy expenditure and improving the stability of head and joint movements, suggesting a complementary relationship between physiological and kinematic factors (Holt et al., 1995). Intramuscular pressure and contractile force are directly related (Sleboda and Roberts, 2020). During walking, intramuscular pressure in the soleus muscle peaks in the late stance phase (Ballard et al., 1998). Without phase synchronization, relatively random systole could lead to variable intramuscular pressures, increasing fluctuations in propulsive force from ankle plantar flexion and potentially destabilizing the walking. However, phase synchronization might stabilize walking by reducing these intramuscular pressure fluctuations across gait cycles. As such, it was hypothesized that stronger CLS would lead to reduced RMS values of upper body acceleration and maxL. Examining this hypothesis can elucidate the kinematic benefits of CLS during normal walking and reveal new aspects of its significance.

## Methods

### Participants

Seven men (age: 23 ± 2 years, height: 172 ± 5 cm, weight: 68 ± 7 kg [average ±standard deviation]) and six women (age: 24 ± 4 years, height: 158 ± 4 cm, weight: 53 ± 9 kg) participated in the study. All participants had no muscular or neurological disorders. Before participation, participants provided written informed consent to participate in the study after receiving a detailed explanation of the study’s purpose, as well as the potential benefits and risks involved. Experimental procedures were conducted in accordance with the principles outlined in the Declaration of Helsinki and received approval from the Local Ethics Committee of the Faculty of the Graduate School of Human Development and Environment at Kobe University (approval number: 661-2).

### Experimental setup

Participants completed a 10-minute treadmill walk at 5.0 km/h. The 5.0 km/h was selected because preliminary experiments confirmed that at this pace, the ratio of the RR interval to step time closely approached 1:1, and participants were able to comfortably walk for a duration of 10 min. They were instructed to refrain from consuming alcohol and caffeine, as well as engaging in heavy physical exercises, starting from the evening prior to the measurements. Initially, the experiments began with determining each participant’s preferred cadence. Starting at 110 steps/min, the cadence was incrementally increased by 1 step/min every 2 steps until participants were instructed to stop the treadmill upon reaching a comfortable cadence. Subsequently, the procedure was repeated starting at 130 steps/min, with the cadence decreasing by 1 step/min every 2 steps until participants reached another comfortable cadence, at which point the treadmill was stopped. The optimal cadence was calculated as the average of the two determined cadences. Thereafter, participants completed a 1-minute treadmill walking exercise at their preferred cadence, regulating their respiration by inhaling and exhaling every 2 steps. During all walking tasks during the experiment, participants matched the metronome sound to the left and right heel contact. Following the treadmill walking exercise, participants rested for 5 min during which they were instructed to inhale and exhale at 2-second intervals. It has been reported that both cardiorespiratory and respiratory-locomotor coupling were extended when breathing rate was fixed to steps (Perry et al., 2020). For this reason, breathing rate was fixed to steps in this study. After the rest period, participants walked for 10 min, maintaining their gaze forward and avoiding unnecessary head movements.

### Data collection

To collect kinematic data, reflective markers were attached to 24 anatomical landmarks over the entire body. The position coordinate values were recorded using a three-dimensional motion capture system with 12 cameras (OptiTrack Flex 3, NaturalPoint, Inc., Corvallis, OR, United States), operating at a sampling frequency of 100 Hz. A gait cycle was defined as right heel contact to the next heel contact. Each step was defined as the moment of heel contact with the corresponding ipsilateral heel.

For electrocardiogram (ECG) data collection, the three electrodes were attached to two rib sites and the space between the clavicles. To reduce motion artifacts, the electrode cables were fixed to the participants using surgical tape. ECG signals were amplified and filtered using a band-pass filter ranging from 0.5 to 120 Hz (EBA-100, Unique Medical, Tokyo, Japan). Electrical signals were sampled at a frequency of 2,000 Hz and stored on a PC hard disk via an analog-to-digital converter (cDAQ-9179, National Instruments, Austin, TX, United States). Following measurements, a Butterworth high-pass filter was applied to eliminate residual motion artifacts (Cutoff frequency = 10 Hz).

### CLS analysis

**FIGURE 1:**
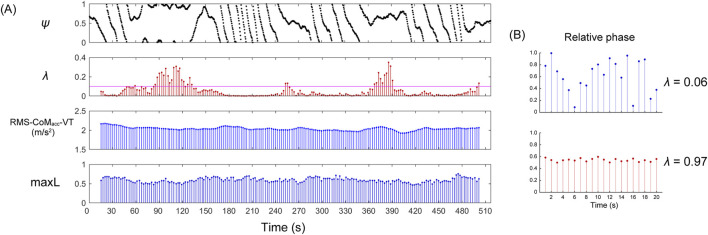
Representative examples of a phase synchronization plot ( ψ ), phase coherence ( λ ), RMS of vertical CoM acceleration (RMS-CoM_acc_-VT), and maximum Lyapunov exponent (maxL). **(A)** The upper plot displays the phase synchronization plot. Parallel segments in which ψ s values are aligned indicate moments when the R-wave consistently occurs within the relative phase. For instance, between 370 and 400 s, ψ is approximately 0.65, indicating that R-waves predominantly appear at approximately 65% of the interval between steps. The *λ* and maxL values are calculated by shifting the window by 5 steps over 60-step intervals; therefore, data near 0 and 510 s are not displayed. For example, the initial *λ* and maxL values are computed from steps one to 60 but are represented by the 30-step mark for convenience. The magenta horizontal line on the phase coherence plot indicates a *λ* value of 0.1. **(B)** Examples of low and high phase coherence. The blue relative phase is spread across the entire phase, indicating low phase coherence. In contrast, the red relative phase is concentrated around 0.55 within the phase, indicating high phase coherence.

The analysis of CLS proceeded through the following steps: first, extraction of cardiac and locomotor rhythms, followed by computation of normalized relative phase, calculation of phase coherence, and comparison with surrogate data. The cardiac rhythm was represented by the RR intervals derived from the ECG, while the locomotor rhythm was represented by the heel strikes of the left and right feet. The first 60 s of data were designated as the adaptation period, and only data recorded after this period were used for analysis. The R-waves were detected using the findpeaks function in MATLAB 2017b (MathWorks, Natick, MA, United States). The r th normalized relative phase within a given locomotor rhythm is expressed as follows (Takeuchi et al., 2014):

$$\psi_{r,h}=\frac{t_{r}-T_{h}}{{T_{h+1}-T}_{h}}$$

where $t_{r}$ denotes the time at which the r th peak of the R-wave occurs, while $T_{h}$ represents the interval between the h th heel strike. A time series data visualizing the timing of the R-wave within the locomotor cycle is referred to as a phase synchronization plot. The plot was generated by plotting $\psi_{r,h}$ as a time series (Figure 1). In the phase synchronization plot, the values of $\psi_{r,h}$ align parallelly, indicating consistent occurrence of heartbeats relative to the locomotion cycle. To evaluate phase synchronization between cardiac and locomotor rhythm, phase coherence (λ) was calculated from the normalized relative phase using the following equation:

$$\lambda ={\left(\frac{1}{N}\sum_{N=1}^{r}\sin\left(\psi_{r,h}\times 2\pi \right)\right)}^{2}+{\left(\frac{1}{N}\sum_{N=1}^{r}\cos\left(\psi_{r,h}\times 2\pi \right)\right)}^{2}$$

The *λ* value is constrained to a range of 0 ≤ *λ* ≤ 1, being maximal for stronger phase locking between the two rhythms and minimal for weaker or no phase locking. The *λ* values over intervals of 60 steps were calculated by incrementally shifting the window by 5 steps each time, thereby capturing the strength of phase synchronization as time series data (Figure 1). In the calculation of the maxL, a minimum of 50 steps is required to account for the effect of experimental noise (Mehdizadeh and Sanjari, 2017). To match the minimum number of steps required for the maxL, the time window for phase synchronization was also set to 60 steps.

To confirm whether the observed CLS occurred by chance, a surrogate data technique was utilized (Nomura et al., 2001). This involved generating surrogate data by randomly shuffling the original order of the step time intervals, leaving the values unchanged and removing only the time series correlation. RR interval intervals were not randomly shuffled. Subsequently, relative phase and phase coherence using surrogate data (*λ*_sur_) were calculated, and the average *λ* and *λ*_sur_ were compared.

### Kinematic data analysis

To evaluate walking smoothness and local dynamical stability, the RMS of the center of mass acceleration (RMS-CoM_acc_) and maxL were calculated. The center of mass position was calculated using the positional coordinate values of the reflective markers. The center of mass acceleration (CoM_acc_) was obtained by taking the second-order derivative of the center of mass position. RMS-CoM_acc_ was calculated in three-dimensional direction (AP: anteroposterior, VT: vertical, ML: mediolateral). When CLS occurs, it is anticipated that the variability of intramuscular pressure between gait cycles decreases. Since intramuscular pressure directly affects muscle contraction force (Sleboda and Roberts, 2020), CLS might influence force output through modulation of intramuscular pressure. Consequently, acceleration—which can reflect force output—was selected as an indicator for stability assessment.

The maxL was derived from the three-dimensional position coordinates of the body’s center of mass (CoM). The state space was constructed using these coordinates. Following recommendations against using a redundant state space that incorporates both position and velocity (Gates and Dingwell, 2009), our analysis focused exclusively on using the positional coordinates of the CoM. The average exponential rate of divergence of neighboring trajectories in the state space was quantified using maxL (Rosenstein et al., 1993). This involved calculating the Euclidean distances between each point in the state space and its nearest neighbor, a process repeated for all data points in the state spaces. The divergence was then calculated as a function of time using the following equation:

$$l\left(i\right)=\langle \ln\left[D_{j}\left(i\right)\right]\rangle /\Delta t$$

where $D_{j}\left(i\right)$ represents the Euclidean distance between the *jth* pair of nearest neighbors after *i* discrete time steps, Δ*t* denotes the sampling period of the time series data, and $\langle \ldots \rangle$ denotes the average overall values of *j*. The maxL was estimated from the slope of the linear fits to the curve and defined as the slope of the linear fits to the divergence curve between 0 and 1 strides (Dingwell and Marin, 2006; Kibushi et al., 2019). A negative maxL indicates convergence among the trajectories of an attractor, whereas a positive maxL indicates divergence. Similar to *λ*, over intervals of 60 steps, RMS-CoM_acc_ and maxL were calculated by incrementally shifting the window by 5 steps each time, capturing the RMS-CoM_acc_ and maxL as time series data (Figure 1).

### Statistics

The *λ* was defined as the high state for *λ* ≥ 0.1 (*λ*_high_) and the low state for *λ* < 0.01 (*λ*_low_). When determining the threshold, the minimum threshold was chosen that was significantly different from the surrogate data and resulted in an average synchronization duration of over 60 s across participants. The *λ*_sur_ was categorized into *λ*_sur_high_ and *λ*_sur_low_ corresponding to the values of *λ*_high_ and *λ*_low_, respectively. Similarly, RR intervals, step length, step time, RMS-CoM_acc_, and maxL were also categorized. Categorized RR intervals, step length, step time, and RMS-CoM_acc_ were averaged within each interval of 60 steps. For each defined state, variables, such as RR intervals, step length, step time, RMS-CoM_acc_, and maxL were computed. The normality of the data was then assessed using the Shapiro–Wilk test. Upon confirming the normal distribution of the data, a paired *t*-test was used to compare the mean values of the RR intervals, step length, step time, RMS-CoM_acc_, and maxL across different states. Another paired *t*-test was conducted to compare the average *λ*_high_/*λ*_low_ and *λ*_sur_high_/*λ*_sur_low_. Cohen’s *d* was used to interpret effect sizes, with values considered small (0.2 < *d* < 0.5), moderate (0.5 < *d* < 0.8), and large (*d* > 0.8). In addition, Bayesian paired *t*-test was performed to quantify the evidence in favor of the null-hypothesis (H0) and alternative hypothesis (H1). As the Bayes factor (BF_10_) deviates from 1, which indicates equal support for H0 and H1, more support is gained for either H0 or H1 (van Doorn et al., 2021). For the evidential strength of H1, BF10 was interpreted as weak (1.00–2.99), moderate (3.00–9.99), and strong (10.00>) (van Doorn et al., 2021). For the evidential strength of H0, BF10 was interpreted as weak (0.33–0.99), moderate (0.10–0.32), and strong (<0.09) (van Doorn et al., 2021). Statistical analyses were performed using JASP version 0.14.1.0 (JASP Team, 2020), with a significance level set at 0.05.

## Results

There was no significant difference observed in the average RR interval between the states (Table 1). Compared to surrogate data, the *λ*_high_ was significantly higher than *λ*_sur_high_, and the *λ*_low_ was significantly lower than *λ*_sur_low_ (Table 1). When comparing the high and low states, *λ*_high_ was significantly higher than *λ*_low_ (Table 1, *p* < 0.001, *t* = 16.312, *d* = 5.437). Average synchronization duration and its standard deviation in the high state was 68 ± 41 s. Each participant’s data on the relative phase and synchronization duration time in strong phase synchronization is represented in Supplementary Material. While step length and step time are significantly lower in the high state (Table 2), the differences are negligible so they can be considered practically equivalent. Furthermore, no significant differences were found in RMS-CoM_acc_ and maxL between the high and low conditions (Table 2).

**TABLE 1:** Descriptive statistics in RR interval, λ and $\lambda_{sur}$ .

	Average	SD	*p*-value	*t*-value	Cohen’s *d*	BF_10_
RR interval-High (s)	0.636	0.083	0.523	0.667	0.222	0.39
RR interval-Low (s)	0.645	0.092				
*λ*_high_	0.146	0.026	<.001	5.576	1.546	10<
*λ*_sur_high_	0.034	0.024				
*λ*_low_	0.004	0.001	<.001	19.142	6.381	10<
*λ*_sur_low_	0.019	0.010				

High: *λ* ≥ 0.1, Low: *λ* < 0.01, *λ*_sur_:*λ* in surrogate data.

**TABLE 2:** Descriptive statistics in step time, step length, RMS-CoM_acc_, maxL.

	Average	SD	*p*-value	*t*-value	Cohen’s *d*	BF_10_
Step time-High (s)	0.708	0.025	0.022	2.849	0.950	3.48
Step time-Low (s)	0.705	0.023				
Step length-High (m)	0.509	0.018	0.022	2.849	0.950	3.48
Step length-Low (m)	0.507	0.017				
RMS-CoM_acc_-AP-High (m/s^2^)	1.084	0.179	0.916	0.109	0.036	0.32
RMS -CoM_acc_-AP-Low (m/s^2^)	1.017	0.136				
RMS -CoM_acc_-VT-High (m/s^2^)	1.834	0.138	0.835	0.216	0.072	0.32
RMS -CoM_acc_-VT-Low (m/s^2^)	1.827	0.165				
RMS -CoM_acc_-ML-High (m/s^2^)	0.357	0.032	0.294	1.123	0.374	0.53
RMS -CoM_acc_-ML-Low (m/s^2^)	0.345	0.026				
maxL-High	0.650	0.108	0.948	0.068	0.023	0.32
maxL-Low	0.627	0.109				

High: *λ* ≥ 0.1, Low: *λ* < 0.01, RMS-CoM_acc_: RMS, of center of mass acceleration, AP, anteroposterior; VT, vertical; ML, mediolateral; maxL, maximum Lyapunov exponent.

## Discussion

This study aimed to determine if RMS-CoM_acc_ and maxL are reduced when phase synchronization between cardiac and locomotor rhythm is strong under regular walking speed. Although *λ*_high_ is significantly higher than *λ*_low_, no significant differences in RMS-CoM_acc_ and maxL can be observed between the high and low states (Table 1). This indicates that at a 5 km/h, strongness of phase synchronization between cardiac and locomotor rhythm does not affect the smoothness of walking or local dynamic stability. This novel finding marks the first investigation into the kinematic significance of CLS.

In this study, the average value of *λ*_high_ was 0.15, lower than the value reported in previous research involving forced synchronization, where *λ* reached 0.56 (Takeuchi et al., 2014). The relatively weak CLS observed in this study may not have reduced RMS-CoM_acc_ and maxL. Compared to surrogate data, *λ*_high_ was significantly higher than *λ*_sur_high_ (Table 1), indicating a weak synchronization state rather than an asynchronous state. Furthermore, previous research has shown that walking with relatively strong CLS results in lower oxygen intake compared to regular walking (Takeuchi et al., 2014). Considering the complementary nature of physiological and kinematic constraints (Holt et al., 1995), it is conceivable that the CLS observed in our study has negligible effects, both physiologically and kinematically. Therefore, the CLS observed at 5.0 km/h is weak, and the benefits of CLS are minimal, potentially lacking significant kinematic effects. To elucidate the kinematic significance, it may be necessary to compare walking with strong CLS to regular walking. Regarding the regulation of CLS, conflict theories have been suggested. Kawahara et al. revealed that the modulation of the CLS originates from the mesencephalic locomotor region in the central nervous system (Kawahara et al., 1993; Kawahara et al., 1994). Conversely, it has been reported that CLS can occur when muscles are passively compressed, indicating that the central nervous activity and afferent nerve activity might be unnecessary (Niizeki, 2005; Niizeki and Saitoh, 2014). Furthermore, the activity of afferent fibers of groups III and IV within skeletal muscles has been shown to mediate the regulation of RR intervals (McWilliam and Yang, 1991). Thus, it may be necessary to modulate muscle activity to induce strong CLS.

The potential impact of weak CLS on kinematic benefits requires an examination of the underlying causes of this phenomenon. One potential reason for the observed weak CLS in this study could be the relatively low exercise load. This research aimed to elucidate the kinematic significance of CLS under conditions resembling daily walking, employing a lower exercise load than previous studies. For example, previous studies set the heart rate at 160 bpm during running (Nomura et al., 2006) and at 120 bpm during walking (Takeuchi et al., 2014), both significantly higher than the 94 bpm observed in this study. Despite anticipating natural synchronization with an average Step: RR ratio close to 1:1, the *λ* value was lower in our study than in previous studies with higher exercise loads. Prior studies examining CLS under relatively low exercise loads have similarly observed weak CLS. For example, Donville et al. (1993) investigated the effect of CLS on oxygen intake during pedaling at the preferred cadence and heat rate of 1:1, finding no physiological benefits among participants (Donville et al., 1993). Niizeki (2005) reported that CLS does not occur at low cuff pressures (Niizeki, 2005), supporting the notion that weak CLS may be induced at lower exercise loads. Additionally, Blain et al. (2009) showed that a workload increase during intense exercise further accentuates CLS (Blain et al., 2009). Therefore, naturally inducing phase synchronization at optimal walking speeds may prove challenging, potentially diminishing both physiological and kinematic benefits.

Walking speed may correlate with weak CLS. The regular or self-selected pace may be a threshold where improvements in kinematic stability become difficult to achieve. Healthy individuals typically exhibit minimal room for improvement in their regular walking speed. Research indicates that kinematic stability is superior at preferred speeds compared to other walking speeds. For instance, the symmetry of lumbar acceleration patterns (Harmonic ratio) is optimal at the preferred walking (Latt et al., 2008). In addition, trunk kinematic variability between strides is minimized at the preferred speed (Dingwell and Marin, 2006), suggesting consistent kinematic stability around the preferred walking speed. Additionally, energy consumption is most efficient at the usual or self-selected walking speed (Cavagna et al., 1976). In a study examining whether biofeedback targeting soleus muscle activity reduces RMS-CoM_acc_ during walking at a 4.5 km/h, only marginal reduction was observed (Kibushi et al., 2024). While further investigation is needed to confirm whether the impact of biofeedback improves at different speeds, this previous study highlights a situation where intervention effects diminish at the preferred walking speed. The preferred speed may pose challenges in detecting intervention effects, potentially contributing to the unchanged smoothness of walking or local dynamic stability.

This study encompasses several limitations. First, the findings are restricted to walking speeds of 5 km/h. Therefore, since this study did not examine lower or higher speeds, further investigation into the effects of different speeds is required. Second, the observed CLS was weak. It exhibited lower phase coherence compared to the data from the previous study, which was conducted under strictly controlled tasks in this study. While this study elucidated the kinematic stability under weak CLS during regular walking, the kinematic stability under stronger CLS remains unclear. Further examination of the effects of forcibly induced synchronization is necessary. The third limitation is the lack of analysis of oxygen intake. While this study successfully elucidated the kinematic significance as its prime objective, it remains unclear whether the weak CLS observed provides physiological advantages. Fourth, the small sample size limits the ability to conclusively determine whether conclusions vary by gender. It is necessary to increase the sample size and devise a method to enforce synchronization at the same walking speed for further verification.

## Conclusion

This study aimed to elucidate the kinematic benefits of CLS during regular walking. However, a comparative analysis between high- and low-phase coherence states, focusing on RMS-CoM_acc_ and maxL, found no significant differences. Hence, CLS appears to have negligible impact on the smoothness of walking or local dynamical stability at a speed of 5.0 km/h.

## Data Availability

The original contributions presented in the study are included in the article/Supplementary Material, further inquiries can be directed to the corresponding author.

## References

[B1] Ballard R. E. Watenpaugh D. E. Breit G. A. Murthy G. Holley D. C. Hargens A. R. (1998). Leg intramuscular pressures during locomotion in humans. J. Appl. Physiol. 84, 1976–1981. 10.1152/jappl.1998.84.6.1976 9609792

[B2] Blain G. Meste O. Blain A. Bermon S. (2009). Time-frequency analysis of heart rate variability reveals cardiolocomotor coupling during dynamic cycling exercise in humans. Am. J. Physiol. - Hear. Circ. Physiol. 296, 1651–1659. 10.1152/ajpheart.00881.2008 19252094

[B3] Bruijn S. M. Bregman D. J. J. Meijer O. G. Beek P. J. van Dieën J. H. (2012). Maximum Lyapunov exponents as predictors of global gait stability: a modelling approach. Med. Eng. Phys. 34, 428–436. 10.1016/j.medengphy.2011.07.024 21856204

[B4] Cavagna G. A. Thys H. Zamboni A. (1976). The sources of external work in level walking and running. J. Physiol. 262, 639–657. 10.1113/JPHYSIOL.1976.SP011613 1011078 PMC1307665

[B5] Constantini K. Stickford A. S. L. Bleich J. L. Mannheimer P. D. Levine B. D. Chapman R. F. (2018). Synchronizing gait with cardiac cycle phase alters heart rate response during running. Med. Sci. Sports Exerc. 50, 1046–1053. 10.1249/MSS.0000000000001515 29240004 PMC6023589

[B6] de Carvalho A. R. Coimbra R. D. S. Thomas E. M. Paz M. C. R. Pellegrini B. Peyré-Tartaruga L. A. (2020). The entrainment frequency of cardiolocomotor synchronization in long-distance race emerges spontaneously at the step frequency. Front. Physiol. 11, 583030. 10.3389/fphys.2020.583030 33613299 PMC7890119

[B7] Dingwell J. B. Marin L. C. (2006). Kinematic variability and local dynamic stability of upper body motions when walking at different speeds. J. Biomech. 39, 444–452. 10.1016/j.jbiomech.2004.12.014 16389084

[B8] Donville J. E. Kirby R. L. Doherty T. J. Gupta S. K. Eastwood B. J. MacLeod D. A. (1993). Effect of cardiac-locomotor coupling on the metabolic efficiency of pedalling. Can. J. Appl. Physiol. 18, 379–391. 10.1139/h93-032 8275051

[B9] Gates D. H. Dingwell J. B. (2009). Comparison of different state space definitions for local dynamic stability analyses. J. Biomech. 42, 1345–1349. 10.1016/j.jbiomech.2009.03.015 19380140 PMC2718682

[B10] Holt K. G. Jeng S. F. Ratcliffe R. Hamill J. (1995). Energetic cost and stability during human walking at the preferred stride frequency. J. Mot. Behav. 27, 164–178. 10.1080/00222895.1995.9941708 12736125

[B11] Iosa M. Fusco A. Morone G. Pratesi L. Coiro P. Venturiero V.-C. (2012). Assessment of upper-body dynamic stability during walking in patients with subacute stroke. J. Rehabil. Res. Dev. 49, 439–450. 10.1682/JRRD.2011.03.0057 22773202

[B12] JASP Team (2020). JASP (version 0.14) [computer software]. Available at: https://jasp-stats.org/faq/how-do-i-cite-jasp/.

[B13] Kawahara K. Yamauchi Y. Niizeki K. Yoshioka T. (1994). Interactions between respiratory, cardiac and stepping rhythms in decerebrated cats: functional hierarchical structures of biological oscillators. Methods Inf. Med. 33, 129–133. 10.1055/s-0038-1634972 8177063

[B14] Kawahara K. Yoshioka T. Yamauchi Y. Niizeki K. (1993). Heart beat fluctuation during fictive locomotion in decerebrate cats: locomotor-cardiac coupling of central origin. Neurosci. Lett. 150, 200–202. 10.1016/0304-3940(93)90535-S 8469421

[B15] Kibushi B. Maekaku K. Kimura T. (2024). Reduced center of mass acceleration during regular walking with electromyography biofeedback. Gait Posture 108, 335–340. 10.1016/J.GAITPOST.2024.01.008 38219328

[B16] Kibushi B. Moritani T. Kouzaki M. (2019). Local dynamic stability in temporal pattern of intersegmental coordination during various stride time and stride length combinations. Exp. Brain Res. 237, 257–271. 10.1007/s00221-018-5422-0 30390101

[B17] Kirby R. L. Nugent S. R. Marlow R. W. MacLeod D. A. Marble A. E. (1989). Coupling of cardiac and locomotor rhythms. J. Appl. Physiol. 66, 323–329. 10.1152/JAPPL.1989.66.1.323 2917937

[B18] Latt M. D. Menz H. B. Fung V. S. Lord S. R. (2008). Walking speed, cadence and step length are selected to optimize the stability of head and pelvis accelerations. Exp. Brain Res. 184, 201–209. 10.1007/s00221-007-1094-x 17717650

[B19] Lockhart T. E. Liu J. (2008). Differentiating fall-prone and healthy adults using local dynamic stability. Ergonomics 51, 1860–1872. 10.1080/00140130802567079 19034782 PMC2892176

[B20] McWilliam P. N. Yang T. (1991). Inhibition of cardiac vagal component of baroreflex by group III and IV afferents. Am. J. Physiol. 260, H730–H734. 10.1152/AJPHEART.1991.260.3.H730 2000968

[B21] Mehdizadeh S. Sanjari M. A. (2017). Effect of noise and filtering on largest Lyapunov exponent of time series associated with human walking. J. Biomech. 64, 236–239. 10.1016/j.jbiomech.2017.09.009 28958634

[B22] Menz H. B. Lord S. R. Fitzpatrick R. C. (2003). Age-related differences in walking stability. Age Ageing 32, 137–142. 10.1093/ageing/32.2.137 12615555

[B23] Mizuike C. Ohgi S. Morita S. (2009). Analysis of stroke patient walking dynamics using a tri-axial accelerometer. Gait Posture 30, 60–64. 10.1016/J.GAITPOST.2009.02.017 19349181

[B24] Niizeki K. (2005). Intramuscular pressure-induced inhibition of cardiac contraction: implications for cardiac-locomotor synchronization. Am. J. Physiol. - Regul. Integr. Comp. Physiol. 288, 645–650. 10.1152/ajpregu.00491.2004 15528394

[B25] Niizeki K. Saitoh T. (2014). Cardiolocomotor phase synchronization during rhythmic exercise. J. Phys. Fit. Sport. Med. 3, 11–20. 10.7600/JPFSM.3.11

[B26] Nomura K. Takei Y. Yanagida Y. (2001). Analysing entrainment of cardiac and locomotor rhythms in humans using the surrogate data technique. Eur. J. Appl. Physiol. 84, 373–378. 10.1007/s004210100382 11417423

[B27] Nomura K. Takei Y. Yoshida M. Yanagida Y. (2006). Phase-dependent chronotropic response of the heart during running in humans. Eur. J. Appl. Physiol. 97, 240–247. 10.1007/s00421-005-0103-7 16506062

[B28] Perry S. Khovanova N. Khovanov I. (2020). “Enhancement of synchronization between physiological signals during exercise: a preliminary investigation,” in 2020 42nd Annual International Conference of the IEEE Engineering in Medicine and Biology Society (EMBC) (IEEE), 461–464. 10.1109/EMBC44109.2020.9175778 33018027

[B29] Rosato A. Larsson M. Rullman E. Dual S. A. (2024). Evidence of spontaneous cardiac-locomotor coupling during daily activities in healthy adults. Front. Physiol. 15, 1394591. 10.3389/fphys.2024.1394591 39253019 PMC11382296

[B30] Rosenstein M. T. Collins J. J. De Luca C. J. (1993). A practical method for calculating largest Lyapunov exponents from small data sets. Phys. D. 65, 117–134. 10.1016/0167-2789(93)90009-P

[B31] Senden R. Savelberg H. H. C. M. Grimm B. Heyligers I. C. Meijer K. (2012). Accelerometry-based gait analysis, an additional objective approach to screen subjects at risk for falling. Gait Posture 36, 296–300. 10.1016/J.GAITPOST.2012.03.015 22512847

[B32] Sleboda D. A. Roberts T. J. (2020). Internal fluid pressure influences muscle contractile force. Proc. Natl. Acad. Sci. U. S. A. 117, 1772–1778. 10.1073/pnas.1914433117 31879350 PMC6983394

[B33] Takeuchi S. Nishida Y. Mizushima T. (2014). Effects of synchronization between cardiac and locomotor rhythms on oxygen pulse during walking. J. Sports Sci. Med. 13, 881–887. 25435781 PMC4234958

[B34] van Doorn J. van den Bergh D. Böhm U. Dablander F. Derks K. Draws T. (2021). The JASP guidelines for conducting and reporting a Bayesian analysis. Psychon. Bull. Rev. 28, 813–826. 10.3758/S13423-020-01798-5 33037582 PMC8219590

[B35] Waters R. L. Lunsford B. R. Perry J. Byrd R. (1988). Energy-speed relationship of walking: standard tables. J. Orthop. Res. 6, 215–222. 10.1002/jor.1100060208 3343627

