# Supplementary material for: Kinematic stability in cardiac locomotor synchronization during regular walking
Source: Front Physiol. 2024 Nov 13;15:1487465. doi: 10.3389/fphys.2024.1487465 (PMC11599197; doi:10.3389/fphys.2024.1487465)
Supplement: Supplementary file 1 [file DataSheet1.pdf]

Supplementary Material

Kinematic stability in cardiac locomotor synchronization during regular walking

Author: Benio Kibushi[1]*

Author Affiliations:

[1] Graduate School of Human Development and Environment, Kobe University, 3-11 Tsurukabuto, Nada, Kobe, Hyogo, JAPAN

Corresponding Author: Benio Kibushi, Ph. D.

E-mail: kibushi.b@ruby.kobe-u.ac.jp

1 Supplementary Data

The supplementary materials contain data for each participant's data on the relative phase and synchronization duration time in strong phase synchronization. Table 1 presents the relative phases, and Table 2 details the synchronization duration in each case. Individual differences were noted in the duration of relative phase and synchronization duration. Although the average relative phase exhibited considerable variability with a large standard deviation, indicating that the data were not robust, it was generally around 0.5, suggesting that the R-wave frequently occurs during the single-leg support phase. Additionally, there was significant variability in the number of synchronization occurrences and synchronization duration.

2 Supplementary Tables

Table 1 Average relative phase during strong phase synchronization in each participant

	Sync1	Sync 2	Sync 3	Sync 4
ID1	0.49±0.28	0.51±0.30		
ID2	0.67±0.13	0.38±0.04	0.50±0.21	
ID3	0.76±0.36	0.64±0.06		
ID4	0.48±0.29			
ID5	0.45±0.03			
ID6	0.47±0.29	0.55±0.29	0.46±0.28	
ID7	0.53±0.07	0.65±0.23		
ID8	0.55±0.30	0.42±0.30	0.50±0.29	0.50±0.28
ID9	0.45±0.31	0.49±0.30	0.47±0.31	
ID10	0.41±0.28	0.47±0.29		
ID11	0.47±0.25			
ID12	0.48±0.28			
ID13	0.46±0.30	0.51±0.28		

Table 2 Synchronization duration during strong phase synchronization in each participant (sec)

	Sync1	Sync 2	Sync 3	Sync 4
ID1	136	32		
ID2	150	33	224	
ID3	93	47		
ID4	142			
ID5	25			
ID6	203	67	95	
ID7	70	51		
ID8	77	52	33	38
ID9	17	42	51	
ID10	52	23		
ID11	18			
ID12	26			
ID13	82	75		
